# Plasma Leptin and Alzheimer Protein Pathologies Among Older Adults

**DOI:** 10.1001/jamanetworkopen.2024.9539

**Published:** 2024-05-03

**Authors:** Seunghoon Lee, Min Soo Byun, Dahyun Yi, Hyejin Ahn, Gijung Jung, Joon Hyung Jung, Yoon Young Chang, Kyungtae Kim, Hyeji Choi, Jeongmin Choi, Jun-Young Lee, Koung Mi Kang, Chul-Ho Sohn, Yun-Sang Lee, Yu Kyeong Kim, Dong Young Lee

**Affiliations:** 1 Department of Psychiatry, Myongji Hospital, Hanyang University College of Medicine, Goyang, Republic of Korea; 2 Department of Neuropsychiatry, Seoul National University Hospital, Seoul, Republic of Korea; 3 Department of Psychiatry, Seoul National University College of Medicine, Seoul, Republic of Korea; 4 Institute of Human Behavioral Medicine, Medical Research Center, Seoul National University, Seoul, Republic of Korea; 5 Interdisciplinary Program of Cognitive Science, Seoul National University College of Humanities, Seoul, Republic of Korea; 6 Department of Psychiatry, Chungbuk National University Hospital, Cheongju, Republic of Korea; 7 Department of Psychiatry, Inje University, Sanggye Paik Hospital, Seoul, Republic of Korea; 8 Department of Neuropsychiatry, SMG-SNU Boramae Medical Center, Seoul, Republic of Korea; 9 Department of Radiology, Seoul National University Hospital, Seoul, Republic of Korea; 10 Department of Nuclear Medicine, Seoul National University College of Medicine, Seoul, Republic of Korea; 11 Department of Nuclear Medicine, SMG-SNU Boramae Medical Center, Seoul, Republic of Korea

## Abstract

**Importance:**

Many epidemiologic studies have suggested that low levels of plasma leptin, a major adipokine, are associated with increased risk of Alzheimer disease (AD) dementia and cognitive decline. Nevertheless, the mechanistic pathway linking plasma leptin and AD-related cognitive decline is not yet fully understood.

**Objective:**

To examine the association of plasma leptin levels with in vivo AD pathologies, including amyloid-beta (Aβ) and tau deposition, through both cross-sectional and longitudinal approaches among cognitively unimpaired older adults.

**Design, Setting, and Participants:**

This was a longitudinal cohort study from the Korean Brain Aging Study for Early Diagnosis and Prediction of Alzheimer Disease. Data were collected from January 1, 2014, to December 31, 2020, and data were analyzed from July 11 to September 6, 2022. The study included a total of 208 cognitively unimpaired participants who underwent baseline positron emission tomography (PET) scans for brain Aβ deposition. For longitudinal analyses, 192 participants who completed both baseline and 2-year follow-up PET scans for brain Aβ deposition were included.

**Exposure:**

Plasma leptin levels as assessed by enzyme-linked immunosorbent assay.

**Main Outcomes and Measures:**

Baseline levels and longitudinal changes of global Aβ and AD-signature region tau deposition measured by PET scans.

**Results:**

Among the 208 participants, the mean (SD) age was 66.0 (11.3) years, 114 were women (54.8%), and 37 were apolipoprotein E ε4 carriers (17.8%). Lower plasma leptin levels had a significant cross-sectional association with greater brain Aβ deposition (β = −0.04; 95% CI, −0.09 to 0.00; *P* = .046), while there was no significant association between plasma leptin levels and tau deposition (β = −0.02; 95% CI, −0.05 to 0.02; *P* = .41). In contrast, longitudinal analyses revealed that there was a significant association between lower baseline leptin levels and greater increase of tau deposition over 2 years (β = −0.06; 95% CI, −0.11 to −0.01; *P* = .03), whereas plasma leptin levels did not have a significant association with longitudinal change of Aβ deposition (β = 0.006; 95% CI, 0.00-0.02; *P* = .27).

**Conclusions and Relevance:**

The present findings suggest that plasma leptin may be protective for the development or progression of AD pathology, including both Aβ and tau deposition.

## Key Points

**Question:**

Are plasma leptin levels associated with baseline and longitudinal changes of the 2 key protein pathologies of Alzheimer disease (AD), amyloid-beta (Aβ) and tau deposition, as measured by positron emission tomography among cognitively normal older adults?

**Findings:**

In this cohort study of 208 cognitively normal older adults, lower plasma leptin levels had a significant cross-sectional association with greater brain Aβ deposition and a significant longitudinal association with greater increase of brain tau deposition over 2 years.

**Meaning:**

The findings based on in vivo human study suggest that plasma leptin may be protective for the development or progression of AD pathology.

## Teaser

This cohort study examines the association of plasma leptin levels with the 2 key protein pathologies of Alzheimer disease, amyloid beta and tau deposition, among cognitively unimpaired older adults.

## Introduction

Alzheimer disease (AD) is a common neurodegenerative disease that is marked by the pathologic accumulation of amyloid-beta (Aβ) and tau proteins in the brain.^1,2^ Although the exact causes of AD are not fully understood yet, it is commonly acknowledged that the development of AD is probably due to a combination of genetic factors, lifestyles, vascular health, and other various risk factors.^3^

Growing evidence indicates that AD is associated with metabolic disorders associated with adipose tissue dysfunction.^4,5^ Leptin, one of the major adipocyte-derived hormones or adipokines, is produced primarily by adipose tissue and plays an important role in regulating appetite, body weight, and energy metabolism.^6^ Leptin is known to be associated with brain physiology through both its regulation of systemic metabolism and its direct effects on the brain.^7^ There is substantial evidence suggesting that leptin operates through central and peripheral mechanisms to regulate systemic metabolism, including glucose use and insulin sensitivity.^8,9^ Furthermore, a deficiency in leptin is closely associated with insulin resistance and hyperglycemia or diabetes,^10,11^ conditions that can affect brain dysfunction.^12,13^ Outside of its role in regulating body weight and systemic metabolism, leptin has been demonstrated to have strong neurotrophic and neuroprotective effects in various animal studies.^14,15,16^ Leptin is also known to modify excitatory synaptic transmission at hippocampal CA1 synapses, enhancing long-term potentiation and improving hippocampal-dependent learning and memory.^17,18,19^

Many epidemiologic studies have suggested that low plasma leptin levels in late life are associated with increased risk of AD dementia and cognitive decline.^20,21,22,23,24^ Nevertheless, the mechanistic pathway linking plasma leptin and AD-related cognitive decline is not fully understood. A recent study demonstrated a significant association between lower plasma leptin levels and decreased cerebrospinal fluid (CSF) Aβ levels.^24^ However, the study had a cross-sectional design, and there is limited information available regarding the longitudinal association between plasma leptin levels and prospective changes of brain Aβ deposition. Furthermore, to our knowledge, no study has investigated the association of leptin with brain tau pathology.

Therefore, the current study aimed to examine the association of plasma leptin levels with in vivo AD pathologies, including Aβ and tau deposition, through both cross-sectional and longitudinal approaches among cognitively unimpaired older adults. We included only cognitively unimpaired individuals to exclude the possibility that the cognitively impaired state itself is associated with plasma leptin (ie, reverse causality) and to focus on the association of leptin with AD pathology. The cognitively impaired state can cause reduced olfactory function and appetite,^25^ inadequate nutrition,^26^ and weight loss,^27^ all of which can be associated with leptin levels.

## Methods

### Participants

This study was conducted as part of an ongoing cohort study called the Korean Brain Aging Study for Early Diagnosis and Prediction of Alzheimer Disease (KBASE),^28^ which began in 2014. Data were collected from January 1, 2014, to December 31, 2020. Participants were recruited from 4 sites (ie, 2 public centers dedicated to dementia prevention and management and 2 memory clinics at 2 university hospitals) in Seoul, South Korea. Individuals potentially meeting the criteria were informed about the study. Those who expressed interest were then invited to undergo an eligibility assessment. In addition, community volunteers were gathered through various means, including online advertisements, posters, brochures distributed at primary recruitment sites, and recommendations from other participants, family members, friends, or acquaintances. As of December 2020, A total of 208 cognitively unimpaired participants who had undergone baseline positron emission tomography (PET) scans for brain Aβ deposition and blood tests were included in the present study. For longitudinal analyses, 192 participants who had completed both baseline and 2-year follow-up PET scans for brain Aβ deposition were included. The participants met the following inclusion criteria: (1) 55 to 90 years of age, (2) Clinical Dementia Rating (CDR) score of 0, and (3) no diagnosis of mild cognitive impairment or dementia. The exclusion criteria were as follows: (1) any serious medical, psychiatric, or neurologic disorder that could affect mental function; (2) any severe communication problem that would render clinical examination or brain scanning difficult; (3) contraindications to magnetic resonance imaging, such as a pacemaker or claustrophobia; (4) absence of a reliable informant; (5) illiteracy, defined as the inability to read; and (6) participation in another clinical trial or treatment with an investigational product. The study was approved by the institutional review board of the Seoul National University Hospital and SNU-SMG Boramae Medical Center, South Korea. All participants provided written informed consent. We adhered to the Strengthening the Reporting of Observational Studies in Epidemiology (STROBE) reporting guideline.

### Clinical Assessment

The participants were comprehensively evaluated by trained psychiatrists at the beginning of the study using the KBASE protocol,^28^ which included the Korean version of the Consortium to Establish a Registry for Alzheimer’s Disease Assessment (CERAD-K) battery.^29,30^ All participants were also systematically assessed for the presence of vascular risk factors such as diabetes, hypertension, dyslipidemia, coronary heart disease, transient ischemic attack, and stroke, based on data collected by trained nurses during systematic interviews with participants and their informants. Each illness (ie, each risk factor) was deemed present if the participant had been diagnosed in a clinic or was taking medications for it at the time of recruitment. The vascular risk factor score (VRS) was calculated based on the number of vascular risk factors.^31^

### Measurement of Serum Leptin Levels

At baseline, blood samples were obtained after an overnight fast via venipuncture in the morning (8-9 am). Plasma leptin levels were measured by enzyme-linked immunosorbent assays using the EZHL-80SK kit (Merck Millipore).

### Assessment of Other Potential Confounders

We systematically evaluated various factors that may play a role in the association between leptin and AD pathologies. Apolipoprotein E (*APOE*) genotyping was performed, and *APOE ε4* (*APOE4*) positivity was defined as the presence of at least 1 ε4 allele. Body mass index (BMI) was calculated using weight in kilograms divided by the height in meters squared and categorized into 3 strata: less than 21 (underweight), 21 to 25 (healthy weight), and more than 25 (overweight), by reference to a previous report.^32^ We made slight adjustments to the definition of underweight in the report (underweight, <20.0; healthy weight, 20.0-24.9; overweight, 25.0-29.9; and obese, ≥30) to enhance statistical reliability, because the proportions of underweight and obese individuals were minimal in our sample when adhering to the definitions from the cited report. Alcohol intake status (categorized as never drinker, former drinker, and current drinker), smoking status (categorized as never smoker, former smoker, and current smoker), and lifetime physical activity were assessed through interviews with nurses. The Lifetime Total Physical Activity Questionnaire^33^ was used to evaluate lifetime physical activity, assigning a metabolic equivalent value to the activity’s intensity according to the Compendium of Physical Activities.^34^

### Measurement of Cerebral Aβ Deposition

All participants received [^11^C] Pittsburgh compound B (PiB) PET scans using a 3.0T Biograph mMR (PET-MR) scanner (Siemens) at baseline. Among them, 192 participants underwent the same scans again at the 2-year follow-up visit. Acquisition of PiB-PET images and preprocessing details were described in a previous report.^35^ To identify regions of interest (ROIs) and assess PiB retention in the frontal, lateral parietal, posterior cingulate-precuneus, and lateral temporal regions, an automatic anatomic labeling algorithm and a region combination method^36^ were used. A global cortical ROI, consisting of the 4 smaller ROIs, was also defined. Global Aβ retention as a standardized uptake value ratio was calculated by dividing the mean values for all voxels of the global cortical ROI by a mean value for a reference region. To analyze the baseline data, the inferior cerebellar gray matter from the spatially unbiased infratentorial template for the cerebellum atlas^37^ was used as a reference region. A participant was considered Aβ positive if global Aβ retention value exceeded 1.21.^38^ For longitudinal analysis, the reference region included the inferior cerebellar gray matter, cerebellar white matter (threshold, 50%), pons, and cerebrum white matter (threshold, 95%, and eroded by 3 voxels).^39,40^

### Measurement of Cerebral Tau Deposition

A subgroup of 76 participants received the initial ^18^F-fluorodeoxyglucose AV-1451 PET scans using a Biograph True Point 40 PET–computed tomography scanner (Siemens). Although the first PiB-PET scan was performed at the baseline visit, the initial AV-1451 PET imaging was initially conducted at a mean (SD) of 2.6 (0.3) years after the baseline visit. Among the participants who underwent the initial AV-1451-PET imaging, 43 individuals received the same scan again 2 years after the initial scan. The methods for AV-1451 PET imaging acquisition and preprocessing have been previously described.^35^ We calculated the AV-1451 standardized uptake value ratio of a priori ROI of the AD-signature region for tau accumulation to estimate cerebral tau deposition. This was a size-weighted mean of the partial volume-corrected uptake by the entorhinal, amygdala, parahippocampal, fusiform, inferior temporal, and middle temporal ROIs.^41,42^ The study used the cerebral hemispheric white matter ROI from FreeSurfer^43^ in the partial volume code^44^ as a reference region according to a literature recommendation for normalizing intensity in longitudinal AV-1451 PET data analysis.^45^

### Statistical Analysis

Statistical analysis took place from July 11 to September 6, 2022. Multiple linear regression analyses were performed to investigate the cross-sectional association between baseline leptin and AD neuroimaging markers. Model 1 included leptin level as an independent variable, each neuroimaging marker as a dependent variable, and age, sex, educational level, and *APOE4* positivity as covariates. Model 2 included VRS and BMI strata as additional covariates as well as the variables and covariates included in model 1. Linear mixed-effects models with random intercepts were applied to examine the associations between baseline plasma leptin and longitudinal change of AD neuroimaging markers over 2 years. Model 1 included baseline leptin, age, sex, educational level, *APOE4* positivity, baseline Aβ (or tau) and their interactions with time. In model 2, we additionally included VRS and BMI strata and their interactions with time. Each participant was set for random intercept, and time was calculated as the number of years from baseline. Data analysis was conducted using jamovi, version 2.2.5.^46^ All *P* values were from 2-sided tests, and results were deemed statistically significant at *P* < .05.

## Results

### Participant Characteristics

Among 208 participants who underwent baseline evaluation (mean [SD] age, 66.0 [11.3] years; 114 women [54.8%] and 94 men [45.2%]), 37 (17.8%) were *APOE4* carriers, and 192 (92.3%) completed the 2-year follow-up PET scans for brain Aβ deposition. The demographic and clinical characteristics of all participants are presented in Table 1 and in eTable 1 and eTable 2 in Supplement 1.

**Table 1.** Participant Characteristics

Variable	Participants (N = 208)
Age at baseline, mean (SD), y	66.0 (11.3)
Female, No. (%)	114 (54.8)
Male, No. (%)	94 (45.2)
Educational level, median (IQR), y	12 (7.0)
*APOE4* carriers, No. (%)	37 (17.8)
Leptin, mean (SD), ng/mL	12.5 (11.1)
Baseline BMI, mean (SD)	24.3 (3.0)
BMI strata, No. (%)	
<21	23 (11.1)
21-25	103 (49.5)
>25	82 (39.4)
Vascular risk factors, No. (%)	
Diabetes mellitus	36 (17.3)
Hypertension	90 (43.3)
Hyperlipidemia	72 (34.6)
Coronary heart disease	10 (4.8)
Stroke	0
Transient ischemic attack	1 (0.5)
VRS, median (IQR)	1 (2)
Alcohol use, No. (%)	
Never	102 (49.0)
Former	23 (11.1)
Current	83 (39.9)
Smoking status, No. (%)	
Never	136 (65.4)
Former	55 (26.4)
Current	17 (8.2)
Lifetime physical activity, MET, median (IQR)	66.2 (55.4)
Cerebral Aβ deposition, SUVR	
Baseline global Aβ retention, median (IQR)	1.12 (0.10)
Baseline Aβ positive (>1.20), No. (%)	41 (19.7)
2-y Change of Aβ, mean (SD) (n = 192)	0.0 (0.09)
Global tau deposition, SUVR	
Baseline tau retention, mean (SD) (n = 76)	1.00 (0.17)
2-y Change of tau, mean (SD) (n = 43)	0.04 (0.15)

Abbreviations: Aβ, amyloid-beta protein; *APOE4*, apolipoprotein E ε4; BMI, body mass index (calculated as weight in kilograms divided by height in meters squared); MET, metabolic equivalent; SUVR, standardized uptake value ratio; VRS, vascular risk score.

### Cross-Sectional Association Between Plasma Leptin and Brain Aβ or Tau Deposition

**Figure 1.**
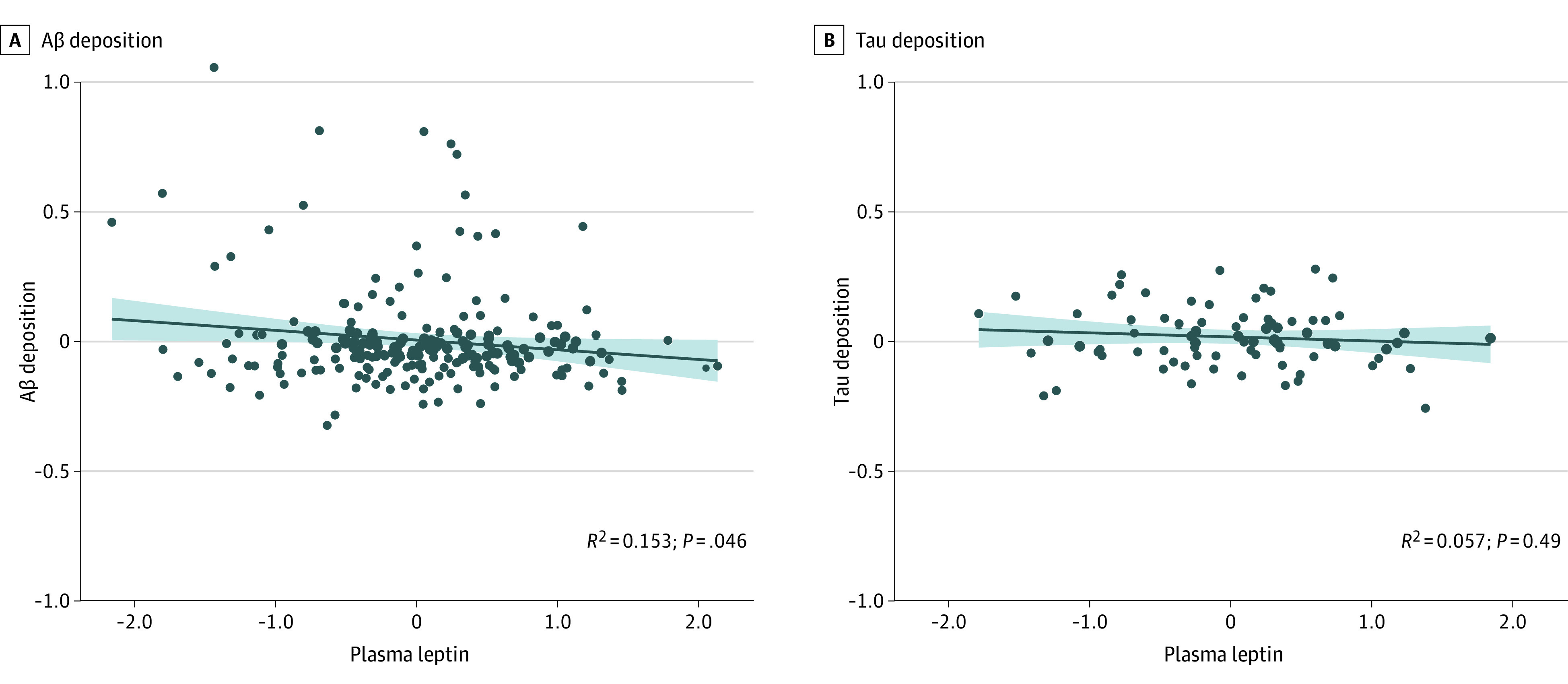
Partial Regression Plots for the Associations Between Plasma Leptin and Alzheimer Disease Biomarkers Partial regression plots show associations between baseline leptin and amyloid-beta (Aβ) deposition (A) and tau deposition (B), adjusted for age, sex, educational level, body mass index strata, apolipoprotein E ε4 positivity, and vascular risk factor scores. The shaded areas indicate the 95% CI.

Baseline leptin had a significant negative correlation with global Aβ deposition at baseline, regardless of the models (Table 2): lower plasma leptin levels were associated with greater brain Aβ deposition (β = −0.04; 95% CI, −0.09 to 0.00; *P* = .046) (Figure 1A). However, there was no significant association between baseline leptin and tau deposition in the AD signature region (β = −0.02; 95% CI, −0.05 to 0.02; *P* = .41) (Table 2 and Figure 1B).

**Table 2.** Association of Baseline Leptin With Baseline Neuroimaging Biomarkers

Variable	Estimated β value (95% CI)	*t* Value	*P* value
Dependent variable: Aβ deposition (n = 208)			
Model 1^a^			
Leptin^b^	−0.04 (−0.07 to 0.00)	−2.13	.04
Model 2^c^			
Leptin^b^	−0.04 (−0.09 to 0.00)	−2.01	.046
Dependent variable: tau deposition (n = 76)			
Model 1^a^			
Leptin^b^	−0.02 (−0.05 to 0.02)	−0.83	.41
Model 2^c^			
Leptin^b^	−0.02 (−0.05 to 0.02)	−0.84	.41

Abbreviations: Aβ, amyloid-beta protein; *APOE4*, apolipoprotein E ε4.

^a^ Adjusted for age, sex, educational level, and *APOE4*.

^b^ Log transformed.

^c^ Adjusted for age, sex, educational level, *APOE4*, body mass index strata, and vascular risk score.

### Longitudinal Association Between Plasma Leptin and the Change in Brain Aβ or Tau Deposition Over 2 Years

The baseline level of plasma leptin did not show a significant association with Aβ deposition change during the 2-year follow-up period, regardless of the models (β = 0.006; 95% CI, 0.00-00.02; *P* = .27). In contrast, there was a significant association between a lower baseline leptin level and a greater increase of tau deposition over 2 years (β = −0.06; 95% CI, −0.11 to −0.01; *P* = .03) (Table 3).

**Table 3.** Association of Baseline Leptin Level With Neuroimaging Biomarker Changes Over a 2-Year Follow-Up Period

Variable	Estimated β value (95% CI)	*t* Value	*P* value
Dependent variable: Aβ deposition			
Model 1^a^			
Baseline leptin × time^b^	0.004 (−0.01 to 0.01)	0.79	.43
Model 2^c^			
Baseline leptin × time^b^	0.006 (0.00 to 0.02)	1.11	.27
Dependent variable: tau deposition			
Model 1^a^			
Baseline leptin × time^b^	−0.06 (−0.10 to −0.02)	−2.66	.01
Model 2^c^			
Baseline leptin × time^b^	−0.06 (−0.11 to −0.01)	−2.18	.03

Abbreviations: Aβ, amyloid-beta protein; *APOE4*, apolipoprotein E ε4.

^a^ Adjusted for age, sex, educational level, *APOE4*, baseline Aβ or tau, and their interactions with time.

^b^ Log transformed.

^c^ Adjusted for age, sex, educational level, *APOE4*, body mass index strata, vascular risk score, baseline Aβ or tau, and their interactions with time.

**Figure 2.**
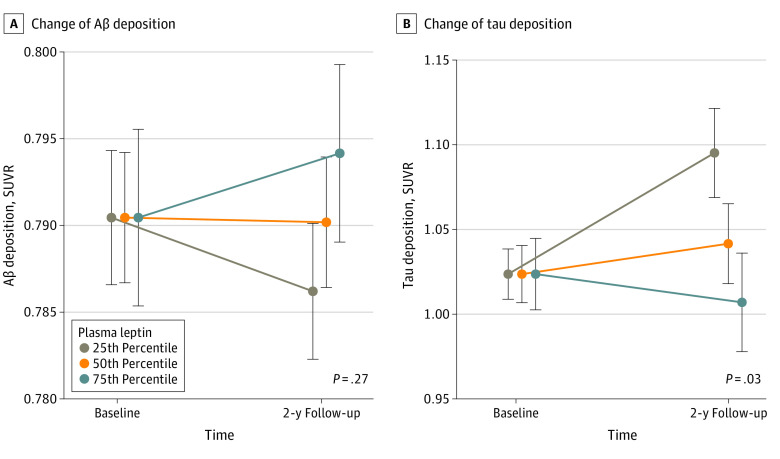
Changes in Alzheimer Disease Biomarkers Over 2 Years According to the Baseline Leptin Level Strata Estimates are from a linear mixed model assessing change in amyloid-beta (Aβ) deposition (A) and tau deposition (B), adjusted for age, sex, educational level, body mass index strata, apolipoprotein E ε4 positivity, vascular risk score, baseline tau or Aβ, and their interactions with time. Error bars indicate SE. SUVR indicates standardized uptake value ratio.

For demonstration, we conducted similar analyses using 3 leptin level strata (below 25th percentile, 25th-75th percentile, and above 75th percentile) instead of leptin level as a continuous variable. The results were very similar, as shown in eTable 3 in Supplement 1 and Figure 2.

## Discussion

The current study first showed that there was a significant cross-sectional association between lower plasma leptin levels and higher brain Aβ deposition. This finding is in line with a previous cross-sectional study that showed that lower plasma leptin levels were associated with lower CSF Aβ concentrations among cognitively impaired individuals.^24^ In contrast to the result from cross-sectional analysis, the result of the longitudinal analysis did not show a significant association between lower baseline leptin levels and prospective increase of brain Aβ deposition. Such a null longitudinal result may be due to the relatively short follow-up period. Given that Aβ accumulation in the brain is a very gradual process,^47^ a 2-year follow-up period might be too short to reveal an association of leptin with Aβ change. As shown in Table 1, there was, on average, no change in Aβ difference between the 2 visits, which supports the idea that the follow-up period might not have been sufficient to detect the association. Given that Aβ accumulates at a relatively consistent rate once an individual crosses the threshold of Aβ positivity until reaching a high level of amyloid burden,^48^ we conducted a subset analysis only for Aβ-positive participants. However, the results remained similar, as shown in eTable 4 in the Supplement 1. Nevertheless, further studies with a larger sample size as well as longer follow-up period are still needed.

In contrast to the results for Aβ, lower baseline plasma leptin levels were associated with greater longitudinal increases of tau deposition. Given the well-known association between brain tau deposition and cognitive decline,^49,50,51^ the finding is generally consistent with previous reports that showed an association between low plasma leptin and increased risk of AD dementia or cognitive decline.^7,20,21,22,23,52^ Meanwhile, the cross-sectional association between plasma leptin and tau deposition was not significant. This null finding may be due to the cognitively intact state of the study participants, in which the level of tau deposition is very low in the neocortex.^53,54^

The mechanism underlying the association of plasma leptin with brain Aβ and tau deposition might be explained by the finding from previous preclinical studies. An experimental study based on both cell cultures and transgenic models demonstrated that leptin reduced the extracellular Aβ level by decreasing γ-secretase activity as well as increasing *APOE*-dependent Aβ uptake.^55^ An animal study showed that treatment with leptin reversed the 27-hydroxycholesterol–induced increase in Aβ and phosphorylated tau by decreasing the levels of BACE1 (beta-site amyloid precursor protein–cleaving enzyme 1) and glycogen synthase kinase-3β (GSK-3β)^56^; GSK-3β is the main tau kinase in the brain and accountable for tau phosphorylation.^57^ Other studies using human cell culture or animal models also showed that leptin treatment reduced tau phosphorylation through inhibition of GSK-3β and 5′-adenosine monophosphate (AMP)–activated protein kinase^58^; AMP-activated protein kinase is associated with tau phosphorylation via GSK-3β.^59^

Given that vascular risk factors—hypertension, diabetes, and hypercholesterolemia in particular—have been associated not only with Aβ or tau deposition,^60,61,62,63,64^ but also with leptin,^65,66,67^ they could potentially confound associations between plasma leptin and AD pathology. Therefore, we controlled for VRS as an additional covariate (in model 2). However, the results remained unchanged even after controlling for VRS.

Regarding BMI, lower or higher BMI categories (ie, underweight or overweight or obese) have been associated with both AD pathology and cognitive impairment.^68,69,70,71^ Body mass index levels are also closely associated with plasma leptin levels,^72^ as observed in our participants (eFigure in Supplement 1). Therefore, BMI levels could be a potential confounder. However, the results were unchanged even after BMI strata were controlled for in model 2. Taken together, these results suggest that the association between plasma leptin and AD pathology was independent of vascular risk factors and BMI, highlighting a specific association between the two. Such a specific association between leptin and AD pathology is in line with some previous reports that showed a significant association between leptin and cognitive decline independent of BMI.^20,22,23,73^

### Limitations

This study has several potential limitations that should be acknowledged. First, we measured the leptin level in plasma, which may not perfectly reflect its cerebral concentration. Further studies including measurement of CSF leptin concentrations are needed. Second, the initial tau PET scan was conducted approximately 2.5 years after leptin measurement at baseline, while the first amyloid PET scan was carried out at baseline. The results remained unchanged when we included the temporal gap as an additional variable in the analysis. However, given a prior report that brain tau accumulated significantly in amyloid-negative, as well as amyloid-positive, healthy older adults over about 2 years of follow-up,^45^ the temporal gap between measurements may have been associated with the outcome. Third, only a smaller subset of 43 participants completed the second tau PET scan. Nevertheless, given that we still found a significant association between leptin and changes in tau deposition, a smaller sample size may not necessarily be a critical concern. Fourth, we included only cognitively unimpaired individuals in order to reduce the possibility that the cognitively impaired state itself is associated with leptin levels. However, given that AD pathology accumulates in the brain decades before the onset of cognitive decline, the possibility that AD pathology is associated with leptin levels (ie, reverse causality) cannot be completely excluded. Some preclinical studies have suggested that Aβ downregulates leptin expression and adversely affects leptin receptor function through allosteric binding.^74,75^ A postmortem brain study also demonstrated that neurofibrillary tangles, which co-localized with leptin receptor–positive cells, were associated with a decrease in active leptin receptors and increase of leptin levels in the brain and cerebral fluid, suggesting that neurofibrillary tangles block circulating leptin from leptin receptors, leading to leptin resistance and decreased leptin signaling.^76^ Fifth, because the recruitment of study participants was not based on random sampling, there is a risk of selection bias that should be considered when interpreting the results.

## Conclusions

The present study is novel in that, to our knowledge, it first revealed the association of plasma leptin levels with both Aβ and tau deposition through longitudinal as well as cross-sectional approaches. The findings, based on an in vivo human study, suggest that plasma leptin may be protective against the development or progression of AD pathology, including both Aβ and tau deposition. In regard to the prevention of AD and related cognitive impairment among older adults, more attention needs to be paid to maintaining appropriate leptin levels.

## Notes

Supplement 1. **eTable 1.** Characteristics of Participants With Baseline Tau PET and Those Without Tau PET in Overall Study Subjects (n=208) **eTable 2.** Characteristics of Participants With Follow-Up Tau PET and Those Without Follow-Up Tau PET Among Participants With Baseline Tau PET (n=76) **eTable 3.** Association of the Baseline Leptin Strata With Neuroimaging Biomarker Changes for 2 Years **eTable 4.** Association of the Baseline Leptin With Aβ Changes for 2 Years in the Group With a Positive Aβ **eFigure.** Comparison of Leptin Levels Between BMI Strata

Supplement 2. **Nonauthor Collaborators.** Members of the Korean Brain Aging Study for Early Diagnosis and Prediction of Alzheimer Disease (KBASE) Research Group

Supplement 3.
Data Sharing Statement
