# Supplementary material for: Plasma Leptin and Alzheimer Protein Pathologies Among Older Adults
Source: JAMA Netw Open. 2024 May 3;7(5):e249539. doi: 10.1001/jamanetworkopen.2024.9539 (PMC11069086; doi:10.1001/jamanetworkopen.2024.9539)
Supplement: Supplement 1. — eTable 1. Characteristics of Participants With Baseline Tau PET and Those Without Tau PET in Overall Study Subjects (n=208) eTable 2. Characteristics of Participants With Follow-Up Tau PET and Those Without Follow-Up Tau PET Among Participants With Baseline Tau PET (n=76) eTable 3. Association of the Baseline Leptin Strata With Neuroimaging Biomarker Changes for 2 Years eTable 4. Association of the Baseline Leptin With Aβ Changes for 2 Years in the Group With a Positive Aβ eFigure. Comparison of Leptin Levels Between BMI Strata [file jamanetwopen-e249539-s001.pdf]

# Supplementary Online Content

Lee S, Byun MS, Yi D, et al. Plasma leptin and Alzheimer protein pathologies among older adults. *JAMA Netw Open.* 2024;7(5):e249539.
doi:10.1001/jamanetworkopen.2024.9539

**eTable 1.** Characteristics of Participants With Baseline Tau PET and Those Without Tau PET in Overall Study Subjects (n=208)

**eTable 2.** Characteristics of Participants With Follow-Up Tau PET and Those Without Follow-Up Tau PET Among Participants With Baseline Tau PET (n=76)

**eTable 3.** Association of the Baseline Leptin Strata With Neuroimaging Biomarker Changes for 2 Years

**eTable 4.** Association of the Baseline Leptin With Aβ Changes for 2 Years in the Group With a Positive Aβ

**eFigure.** Comparison of Leptin Levels Between BMI Strata

This supplementary material has been provided by the authors to give readers additional information about their work.

© 2024 Lee S et al. *JAMA Network Open.*

**eTable 1.** Characteristics of Participants With Baseline Tau PET and Those Without Tau PET in Overall Study Subjects (n=208)

| Variable | Baseline Tau PET (+) | Baseline Tau PET (-) | p-value |
|---|---|---|---|
| No. of individuals | 76 | 132 | |
| Age at baseline, year (mean ± SD) | 66.9 ± 10.8 | 65.5 ± 11.6 | 0.38 |
| Sex, No. (%) | | | 0.12 |
| Female | 47 (61.8) | 67 (50.8) | |
| Male | 29 (38.2) | 65 (49.2) | |
| Education, year, median (IQR) | 12 (7.0) | 12 (7.0) | 0.57 |
| APOE4 carriers, No. (%) | 14 (18.4) | 23 (17.4) | 0.86 |
| Leptin (ng/ml) | 12.1 ± 9.4 | 12.8 ± 12.0 | 0.71 |
| Baseline BMI, kg/m$^2$ (mean ± SD) | 24.4 ± 2.8 | 24.2 ± 3.2 | 0.55 |
| BMI strata, No. (%) | | | 0.27 |
| BMI < 21 | 5 (6.6) | 18 (13.6) | |
| 21 ≤ BMI ≤ 25 | 41 (53.9) | 62 (47.0) | |
| BMI > 25 | 30 (39.5) | 52 (39.4) | |
| Vascular risk factor, No. (%) | | | |
| Diabetes mellitus | 19 (25.0) | 17 (12.9) | 0.03 |
| Hypertension | 32 (42.1) | 58 (43.9) | 0.80 |
| Hyperlipidemia | 27 (35.5) | 45 (34.1) | 0.83 |
| Coronary heart disease | 4 (5.3) | 6 (4.5) | 0.82 |
| Stroke | 0 (0) | 0 (0) | NA |
| TIA | 1 (1.3) | 0 (0) | 0.19 |
| VRS, median (IQR) | 1 (2.0) | 1 (2.0) | 0.32 |
| Alcohol use, No. (%) | | | 0.24 |
| Never | 43 (56.6) | 59 (44.7) | |
| Former | 8 (10.5) | 15(11.4) | |
| Drinker | 26 (33.3) | 58 (43.9) | |
| Smoking status, No. (%) | | | 0.34 |
| Never | 54 (71.1) | 82 (62.1) | |
| Former | 18 (23.7) | 37 (28.0) | |
| Smoker | 4 (5.3) | 13 (9.8) | |
| Lifetime physical activity, MET, median (IQR) | 62.7 (43.6) | 69.1 (64,1) | 0.43 |
| Cerebral Aβ deposition, SUVR | | | |
| Baseline global Aβ retention, median (IQR) | 1.13 (0.14) | 1.11 (0.08) | <0.01 |
| Baseline Aβ positive (>1.20), No, (%) | 23 (30.3) | 18 (13.6) | <0.01 |
| Global Tau deposition, SUVR | | | |
| Baseline Tau retention, (mean ± SD), (n=76) | 1.00 (0.17) | NA | NA |

Abbreviations: Aβ, β-amyloid protein; APOE4, apolipoprotein E ε4; IQR, Interquartile range; MET, metabolic equivalent; SD, standard deviation; SUVR, standardized uptake value ratio; VRS, vascular risk score.

© 2024 Lee S et al. *JAMA Network Open.*

**eTable 2.** Characteristics of Participants With Follow-Up Tau PET and Those Without Follow-Up Tau PET Among Participants With Baseline Tau PET (n=76)

| Variable | Follow-up Tau PET (+) | Follow-up Tau PET (-) | p-value |
|---|---|---|---|
| No. of individuals | 43 | 33 | |
| Age at baseline, year (mean ± SD) | 69.0 ± 7.5 | 64.5 ± 13.4 | 0.14 |
| Sex, No. (%) | | | 0.07 |
| Female | 22 (51.2) | 24 (72.7) | |
| Male | 21 (48.8) | 9 (27.3) | |
| Education, year, median (IQR) | 12 (9.0) | 12 (4.0) | 0.31 |
| APOE4 carriers, No. (%) | 7 (16.3) | 7 (21.2) | 0.46 |
| Leptin (ng/ml) | 12.8 ± 9.2 | 11.4 ± 9.7 | 0.51 |
| Baseline BMI, kg/$m^2$ (mean ± SD) | 24.4 ±2.5 | 24.5 ± 3.2 | 0.85 |
| BMI strata, No. (%) | | | 0.81 |
| BMI < 21 | 3 (7.0) | 3 (9) | |
| 21 ≤ BMI ≤ 25 | 24 (55.8) | 16 (48.5) | |
| BMI > 25 | 16 (37.2) | 14 (42.4) | |
| Vascular risk factor, No. (%) | | | 0.70 |
| Diabetes mellitus | 9 (20.9) | 9 (27.2) | 0.43 |
| Hypertension | 20 (46.5) | 11 (33.3) | 0.28 |
| Hyperlipidemia | 16 (37.2) | 11 (33.3) | 0.79 |
| Coronary heart disease | 2 (4.7) | 1 (3.0) | 0.83 |
| Stroke | 0 (0) | 0 (0) | NA |
| TIA | 1 (2.3) | 0 (0) | 0.36 |
| VRS, median (IQR) | 1 (2) | 1 (2) | 0.48 |
| Alcohol use, No. (%) | | | 0.34 |
| Never | 22 (51.2) | 20 (60.6) | |
| Former | 7 (16.3) | 2 (6.1) | |
| Drinker | 14 (32.6) | 11 (33.3) | |
| Smoking status, No. (%) | | | 0.41 |
| Never | 28 (65.1) | 27 (81.8) | |
| Former | 13 (30.2) | 5 (15.2) | |
| Smoker | 2 (4.7) | 1 (3.0) | |
| Lifetime physical activity, MET, median (IQR) | 66.6 (40.0) | 51.9 (43.3) | 0.32 |
| Cerebral Aβ deposition, SUVR | | | |
| Baseline global Aβ retention, median (IQR) | 1.12 (0.11) | 1.15 (0.39) | 0.43 |
| Baseline Aβ positive (>1.20), No, (%) | 11 (25.6) | 12 (36.4) | 0.27 |
| Global Tau deposition, SUVR | | | |
| Baseline Tau retention, (mean ± SD) | 1.02 (0.12) | 1.01 (0.11) | 0.96 |

Abbreviations: Aβ, β-amyloid protein; APOE4, apolipoprotein E ε4; IQR, Interquartile range; MET, metabolic equivalent; SD, standard deviation; SUVR, standardized uptake value ratio; VRS, vascular risk score.

© 2024 Lee S et al. *JAMA Network Open.*

**eTable 3.** Association of the Baseline Leptin Strata With Neuroimaging Biomarker Changes for 2 Years

| | **Estimate (95% CI)** | ***t* value** | ***p* value** |
|---|---|---|---|
| **Dependent variable: Aβ retention** | | | |
| Baseline Leptin[a] strata x time | 0.00 (-0.02 to 0.03) | 0.27 | .79 |
| **Dependent variable: Tau deposition** | | | |
| Baseline Leptin[a] strata x time | -0.12 (-0.24 to -0.01) | -2.14 | .04 |

Abbreviations: Aβ, β-amyloid protein; APOE, apolipoprotein e; CI, confidence interval.
Adjusted for age, sex, education, APOE e4, BMI strata, vascular risk score, baseline Aβ or Tau and their interactions with time.
[a] log transformed.

© 2024 Lee S et al. *JAMA Network Open.*

**eTable 4.** Association of the Baseline Leptin With Aβ Changes for 2 Years in the Group With a Positive Aβ

| | **Estimate (95% CI)** | ***t* value** | ***p* value** |
|---|---|---|---|
| **Dependent variable: Aβ deposition** | | | |
| Model 1[a] | | | |
| Baseline Leptin[c] x time | -0.01 (-0.02 to 0.01) | -0.54 | .59 |
| Model 2[b] | | | |
| Baseline Leptin[c] x time | 0.01 (0 to 0.03) | 1.46 | .15 |

Abbreviations: Aβ, β-amyloid protein; APOE4, apolipoprotein E ε4; CI, confidence interval.

[a] Adjusted for age, sex, education, APOE4, baseline Aβ and their interactions with time.

[b] Adjusted for age, sex, education, BMI strata, APOE4, vascular risk score, baseline Aβ, and their interactions with time.

[c] log transformed.

© 2024 Lee S et al. *JAMA Network Open.*

**eFigure.** Comparison of Leptin Levels Between BMI Strata

Note. By one-way ANOVA with Turkey's post-hoc test.

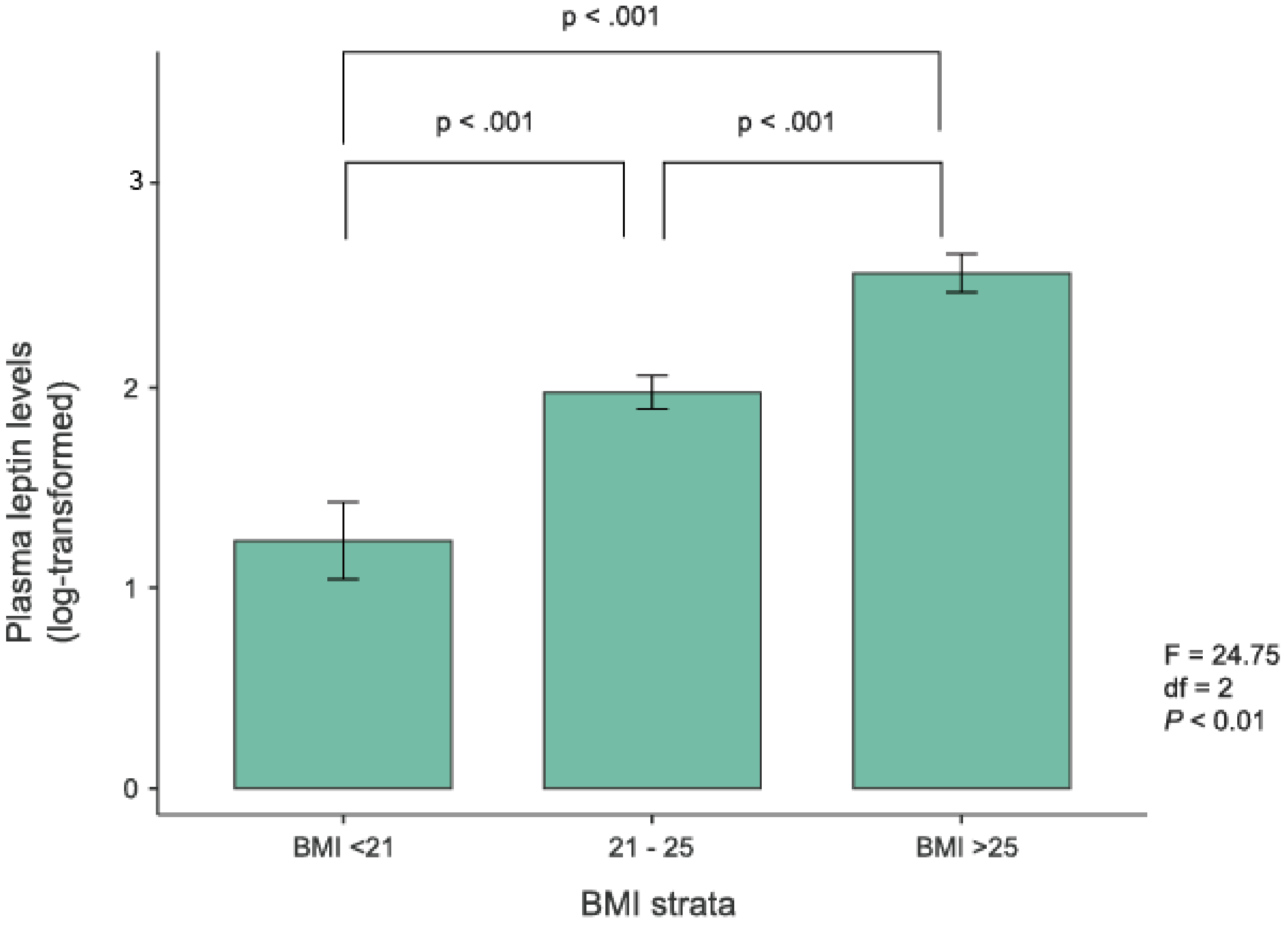


© 2024 Lee S et al. *JAMA Network Open.*
