# Supplementary material for: Plasma Leptin and Alzheimer Protein Pathologies Among Older Adults
Source: JAMA Netw Open. 2024 May 3;7(5):e249539. doi: 10.1001/jamanetworkopen.2024.9539 (PMC11069086; doi:10.1001/jamanetworkopen.2024.9539)
Supplement: Supplement 2. — Nonauthor Collaborators. Members of the Korean Brain Aging Study for Early Diagnosis and Prediction of Alzheimer Disease (KBASE) Research Group [file jamanetwopen-e249539-s002.pdf]

Supplemental Online Content: Nonauthor Collaborators
*First name, last name, and suffix (if applicable) are required and will appear in PubMed.

***Group Name(s): Korean Brain Aging Study for Early Diagnosis and Prediction of Alzheimer Disease (KBASE)**
[illegible]

***First Name and Middle Initial(s)**	***Last Name**	***Suffix (eg, Jr, III)**	Academic Degrees	Institution	Location (city, state/province, country)	Role or Contribution, eg, chair, principal investigator
Inhee	Mook		PhD	Seoul National University	Seoul, Republic of Korea	Core PI Biomarker
Murim	Choi		PhD	Seoul National University	Seoul, Republic of Korea	Core PI Genetic
Yu Jin	Lee		MD, PhD	Seoul National University	Seoul, Republic of Korea	Core PI sleep
Seokyung	Hahn		PhD	Seoul National University	Seoul, Republic of Korea	Core PI Biostatistics
Hyun Jung	Kim		MD	Changsan Convalescent Hospital		Co-investigator
Mun Young	Chang		MD, PhD	Chung-Ang University College of Medicine	Seoul, Republic of Korea	Co-investigator
Na Young	Han		MD	Dongrae Hospital	Pusan, Republic of Kore	Co-investigator
Jisoo	Pae		MD, PhD	Genome & Company		Co-investigator
Hansoo	Park		MD, PhD	Genome & Company		Co-investigator
Jee Wook	Kim		MD, PhD	Hallym University Dongtan Sacred	Hwaseong-si, Gyeonggi-	Co-investigator
Jong-Min	Lee		PhD	Hanyang University	Seoul, Republic of Korea	Co-investigator
Dong Woo	Lee		MD, PhD	Inje University Sanggye Paik Hos	Seoul, Republic of Korea	Co-investigator
Bo Kyung	Sohn		MD, PhD	Inje University, Sanggye Paik Hos	Seoul, Republic of Korea	Co-investigator
Seok Woo	Moon		MD, PhD	Konkuk University Chungju Hospi	Chungju,Chungcheongb	Co-investigator
Hyewon	Baek		MD	Gyeonggi Provincial Hospital for t	Gyeonggi-do, Republic c	Co-investigator
Yoon-Keun	Kim		MD, PhD	MD Healthcare Inc.		Co-investigator
Jong-Won	Kim		MD, PhD	Samsung Medical Center	Seoul, Republic of Korea	Co-investigator
Seung-Ho	Ryu		MD, PhD	School of Medicine Konkuk Unive	Seoul, Republic of Korea	Co-investigator
Shin Gyeom	Kim		MD, PhD	Soonchunhyang University Hospit	Bucheon, Gyeonggi-do,	Co-investigator
Jong Inn	Woo		MD, PhD	Seoul National University	Seoul, Republic of Korea	Co-investigator
Sang Eun	Kim		MD, PhD	Seoul National University Bundan	Seongnam-si, Gyeonggi-	Co-investigator
Gi Jeong	Cheon		MD, PhD	Seoul National University Hospital	Seoul, Republic of Korea	Co-investigator
Koung Mi	Kang		MD, PhD	Seoul National University Hospital	Seoul, Republic of Korea	Co-investigator
Jee-Eun	Park		MD, PhD	Seoul National University Hospital	Seoul, Republic of Korea	Co-investigator
Hyeong Gon	Yu		MD, PhD	SMG-SNU Boramae Medical Cen	Seoul, Republic of Korea	Co-investigator
Hyo Jung	Choi		MD, PhD	SMG-SNU Boramae Medical Cen	Seoul, Republic of Korea	Co-investigator

Supplemental Online Content: Nonauthor Collaborators
*First name, last name, and suffix (if applicable) are required and will appear in PubMed.

*First Name and Middle Initial(s)	*Last Name	*Suffix (eg, Jr, III)	Academic Degrees	Institution	Location (city, state/province, country)	Role or Contribution, eg, chair, principal investigator
Young Min	choe		MD, PhD	Hallym University Dongtan Sacred	Hwaseong-si, Gyeonggi-	Co-investigator
Kwangsoo	Kim		PhD	Seoul National University Hospital	Seoul, Republic of Korea	Co-investigator
So Yeon	Jeon		MD, PhD	Chungnam National University Hospital	Daejeon, Republic of Ko	Co-investigator
Woo Jin	Kim		MD, PhD	Seoul National University Hospital	Seoul, Republic of Korea	Co-investigator
Kang	Ko		MD	National Health Insurance Service	Ilsan si, Gyeonggi-do, Re	Co-investigator
Jun Ho	Lee		MD, PhD	Seoul National University Hospital	Seoul, Republic of Korea	Research fellow
Sung Wook	Park		MD, PhD	Seoul National University Hospital	Seoul, Republic of Korea	Research fellow
Gijung	Jung		RN, PhD	Seoul National University Hospital	Seoul, Republic of Korea	Research Coordinator
Haejung	Joung		MS	Seoul National University Hospital	Seoul, Republic of Korea	Psychologist
HyeJin	Ann		RN	Seoul National University	Seoul, Republic of Korea	Psychologist
Han Na	Lee		RN	Seoul National University Hospital	Seoul, Republic of Korea	Research Coordinator
Joon Hyung	Jung		MD	Chungbuk National University Hos	Cheongju-si, Chungcheo	Co-investigator
Gihwan	Byeon		MD	Kangwon National University Hos	Chuncheon-si, Gangwon	Co-investigator
Kiyoung	Sung		MD	Seoul National	Seoul, Republic of Korea	Research fellow
Dong Kyun	Han		MD	Seoul National University Hospital	Seoul, Republic of Korea	Research fellow
Seung Min	Han		MD	Seoul National University Hospital	Seoul, Republic of Korea	Research fellow
Min Jung	Kim		MD	Seoul National University Hospital	Seoul, Republic of Korea	Research fellow
Min Jae	Kim		MD	Soonchunhyang University Hospital	Seoul, Republic of Korea	Co-investigator
Nayeong	Kong		MD, PhD	Keimyung University Dongsan Ho	Daegu, Republic of Kore	Co-investigator
Seo Hee	Park		MD	Seoul National University Hospital	Seoul, Republic of Korea	Co-investigator
Mimi	Kim		RN, PhD	Seoul National University Hospital	Seoul, Republic of Korea	Research coordinator
Woojin	Cha		MS	Seoul National University Hospital	Seoul, Republic of Korea	Psychologist
Hyeryeon	Yeom		MS	Seoul National University Hospital	Seoul, Republic of Korea	Psychologist
Yoon Young	Chang		MD	Inje University Sanggye Baek Hos	Seoul, Republic of Korea	Co-investigator

Supplemental Online Content: Nonauthor Collaborators

*First name, last name, and suffix (if applicable) are required and will appear in PubMed.

***First Name and Middle Initial(s)**	***Last Name**	***Suffix (eg, Jr, III)**	Academic Degrees	Institution	Location (city, state/province, country)	Role or Contribution, eg, chair, principal investigator	
Musung	Keum		MD	Seoul National University Hospital	Seoul, Republic of Korea	Research fellow	
Min Jeong	Kim		RN	Seoul National University	Seoul, Republic of Korea	Research coordinator	
Donghee	Kim		RN	Seoul National University Hospital	Seoul, Republic of Korea	Research coordinator	
Kyungtae	Kim		MD	Seoul National University Hospital	Seoul, Republic of Korea	Psychologist	
Jeongmin	Choi		MD	Seoul National University Hospital	Seoul, Republic of Korea	Research fellow	
Hye Ji	Choi		MD	Seoul National University Hospital	Seoul, Republic of Korea	Research fellow	
Han Sol	Bae		RN	Seoul National University Hospital	Seoul, Republic of Korea	Research coordinator	
Dohyun	Woo		MS	Seoul National University Hospital	Seoul, Republic of Korea	Psychologist	
Seunghyuk	Ha		MS	Seoul National University Hospital	Seoul, Republic of Korea	Psychologist	
