# Supplementary material for: Plasma Leptin and Alzheimer Protein Pathologies Among Older Adults
Source: JAMA Netw Open. 2024 May 3;7(5):e249539. doi: 10.1001/jamanetworkopen.2024.9539 (PMC11069086; doi:10.1001/jamanetworkopen.2024.9539)
Supplement: Supplement 3. — Data Sharing Statement [file jamanetwopen-e249539-s003.pdf]

Data Sharing Statement

Lee. Plasma Leptin and Alzheimer Protein Pathologies Among Older Adults. *JAMA Netw Open*. Published May 03, 2024. doi:10.1001/jamanetworkopen.2024.9539

Data
Data available: No

Additional Information
Explanation for why data not available: The datasets generated and analyzed during the present study are not publicly available, owing to ethics considerations and privacy restrictions. Data might be obtained from the corresponding author after approval by the Institutional Review Board of the Seoul National University Hospital, South Korea.
